# Absence of ductal hyper-keratinization in Mouse age-related meibomian gland dysfunction (ARMGD)

**DOI:** 10.18632/aging.100615

**Published:** 2013-11-18

**Authors:** Geraint J. Parfitt, Yilu Xie, Mikhail Geyfman, Donald J. Brown, James V. Jester

**Affiliations:** Gavin Herbert Eye Institute, University of California, Irvine, Irvine, CA 92697-4390, USA

**Keywords:** ICT, 3-D reconstruction, immunofluorescence, Meibomian Gland, dry eye disease

## Abstract

Meibomian gland dysfunction (MGD) is frequent with aging and is the primary cause of dry eye disease, the most prevalent ocular complaint. We used a novel 3-D reconstruction technique, immunofluorescent computed tomography (ICT), to characterize meibomian gland keratinization and cell proliferation in a mouse model of age-related meibomian gland dysfunction (ARMGD). To visualize the changes associated with ARMGD, 5-month and 2-year old mouse eyelids were 3-D reconstructed by ICT using antibodies to cytokeratin (CK) 1, 5 and 6 and the proliferation marker Ki67. We quantified total gland, ductal and lipid volume from the reconstructions, observing a dramatic decrease in old glands. In young glands, proliferative ductules suggest a potential site of acinar progenitors that were found to be largely absent in aged, atrophic glands. In the aged mouse, we observed an anterior migration of the mucocutaneous junction (MCJ) and an absence of hyper-keratinization with meibomian gland atrophy. Thus, we propose that changes in the MCJ and glandular atrophy through a loss of meibocyte progenitors are most likely responsible for ARMGD and not ductal hyper-keratinization and gland obstruction.

## INTRODUCTION

Dry eye disease (DED) affects an estimated 21 million individuals in the United States and the incidence increases with age [1-4]. The disease can be exacerbated by contact lens wear and low humidity environments; severely limiting reading, driving and performance at computer display terminals. If untreated, DED can increase the risk and severity of visual problems including microbial keratitis and corneal injury [5-8]. Individuals suffering from DED frequently complain of ocular surface irritation, photophobia and blurred vision leading to reduced quality of life and productivity. While DED can have multiple etiologies, recent studies suggest that dysfunction of the lipid secreting glands of the eyelid tarsal plate, i.e. the meibomian glands, is the major cause of DED. Meibomian gland dysfunction (MGD) in the form of gland dropout and changes in lipid quality can be detected in over 85% of DED patients evaluated in clinical-based studies [3, 9]. Current treatment of MGD is primarily palliative and limited to eyelid hygiene with warm compresses and anti-microbial/anti-inflammatory therapy [10]. Therefore, a greater understanding of the mechanisms that initiate age-related MGD (ARMGD) is required to develop more effective therapies to the disease.

Meibomian glands are holocrine, modified sebaceous glands that secrete lipids (meibum) onto the ocular surface, where they increase tear-film stability, decrease aqueous tear evaporation and provide a smooth optical surface [11-13]. In ARMGD, abnormal secretion of tear film lipids leads to the increased evaporation of tears causing increased tear osmolarity, release of inflammatory cytokines and the symptoms of DED [13-16]. The presence of MGD is detected by the clinical examination of the eyelids, which show gland dropout and the expression of a ‘tooth paste-like’ excreta in severe cases. It has been proposed that development of ARMGD involves obstruction of the gland by hyper-keratinization of the duct and gland orifice leading to plugging, cystic dilation and atrophy associated with changes in lipid quality [11, 17-21].

Evidence for obstructive MGD in human patients has been supported by the identification of ‘keratotic’ clusters of squamous cells detected in MGD excreta [22], and histopathological evidence showing isolated regions of abnormal keratinization, ductal dilation and enlarged acini [23]. While a recent study of gene expression patterns of MGD glands has detected increased expression of genes associated with keratinization [24], analysis of proteins from excreta of MGD subjects failed to detect cytokeratin (CK) 1/10, the biomarkers for epidermal keratinization, while there was a general increase in other CKs associated with non-keratinized epithelium [25]. Recently, meibomian gland dropout has been documented in wild-type mice over 1 year of age [26, 27]. Since meibomian gland dropout is highly correlated with changes in lipid quality and frequently observed in human subjects over the age of 50[1, 4, 28-30], study of this mouse model may help in identifying underlying pathogenic mechanisms of ARMGD and suggest novel and more effective therapeutic strategies for this widespread clinical problem.

Immunofluorescent Computed tomography (ICT) is a novel technique based on butyl-methyl methacrylate (BMMA) embedding that allows for repeated antibody-based staining on serial tissue sections cut in the range of ultra-thin (0.1μm) to semi-thin (5μm) thickness while maintaining excellent morphological preservation of tissue[31]. This enables 3-D reconstruction of multiple antigens with more reliable immunostaining and higher axial resolution across a large volume (>1mm^3^) than possible with conventional immunohistochemistry methods. We used ICT to characterize the distribution of epithelial CK proteins (1, 5 and 6) in a young, healthy mouse eyelid and an old mouse eyelid with ARMGD to assess the connection between keratinization, gland plugging and acinar atrophy with aging. CKs were chosen based on a previous study which examined their distribution at the mucocutaneous junction of the eyelid [32]. Results obtained here further demonstrate how ICT can be used for probing tissue structure and function from the macro (whole meibomian gland) to the micro (meibocyte) level where conventional histopathologic and immunofluorescent approaches are limited due to sampling error during repetitive antigen probing and 3-D reconstruction.

## RESULTS

### Effect of Aging on Eyelid and Meibomian Gland Architecture

**Figure 1:**
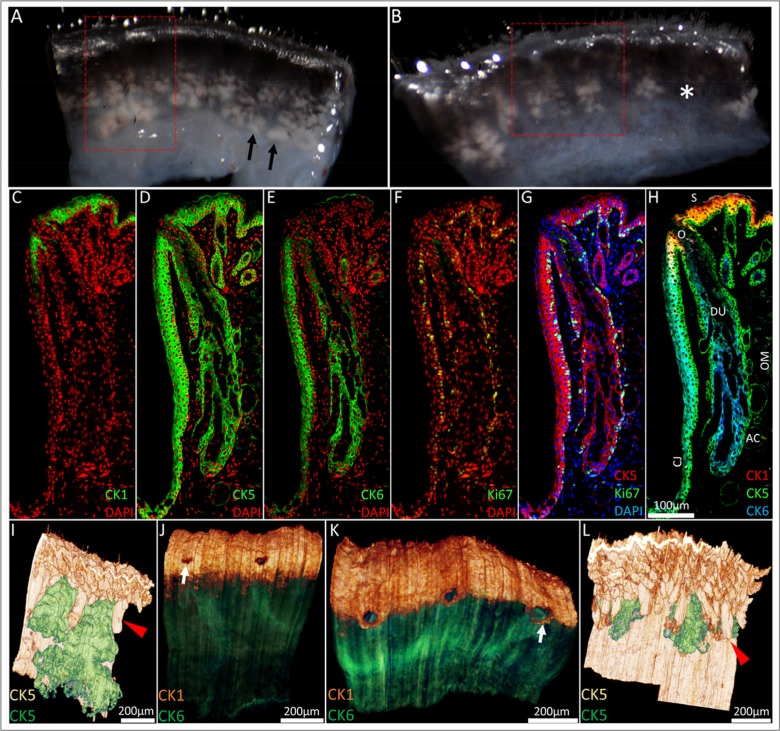
From the macroscopic to the microscopic: ICT reconstruction of the young and aged mouse eyelid. (**A**) Dissecting microscope images of a 5-month old and (**B**) 2-year old mouse eyelid. Meibomian glands (black arrowheads) were identified as whitish tissue underneath transparent conjunctiva and gland dropout was observed in the older mouse eyelids (asterisk). Eyelids were embedded in BMMA and serially sectioned; the red box highlights the areas 3-D reconstructed through sequential immunostaining, as shown in C-H (**C**) A 5month old mouse eyelid 2μm BMMA section stained with CK1 and DAPI before antibody elution with glycine HCl and subsequent immunostaining with (**D**) CK5, (**E**) CK6 and (**F**) Ki67 antibodies. The resulting overlays (**G** and **H**) show the meibomian gland parallel to the conjunctiva (CJ) and embedded in the orbicularis muscle (OM). The meibomian gland duct (DU) and orifice (O) are CK5^+^/CK6^+^, whereas acinar (AC) and skin (S) epithelium are CK5^+^/CK6^−^. CK5-based reconstructions of a (**I**) 5month and (**L**) 2year old mouse eyelid visualised the hair follicle (red arrowheads) and meibomian gland epithelia (green), confirming the truncation of meibomian glands with age. In 3-D, CK1 staining at the lid margin of (**J**) young and (**K**) old lids revealed a loss of a layer of epidermal cells posterior to meibomian gland orifices in the older lid. This caused an anterior shift of the mucocutaneous junction (white arrows) to the level of the meibomian gland orifices.

Prior to BMMA embedding and immunofluorescence staining to compare the effects of aging, excised eyelids from a 5-month and 2-year old mouse were imaged under a dissecting microscope at 43× magnification (Fig. 1A and B, respectively) and the meibomian glands were identified as a whitish tissue underlying the palpebral conjunctiva (black arrows). Loss of lipid containing acinar tissue consistent with meibomian gland dropout was observed in the 2-year old (Fig 1B, asterisk) and not the younger 5-month old mouse eyelid tissue. Next, BMMA serial sections cut at 2μm were sequentially immunostained with CK1, 5, 6 and Ki67 and 0.64mm × 1.14mm × 1.25mm of the 5-month old mouse eyelid (Fig 1A, red box) and 0.58mm × 0.80mm × 1.1mm of the 2-year old mouse eyelid (Fig 1B, red box) were 3-D reconstructed by ICT protocol. The typical CK1, 5, 6 and Ki67 immuno-staining patterns of a young mouse eyelid are shown in Figure 1C-H. In the 5-month old mouse eyelid CK5 reconstruction (Fig. 1I), meibomian glands (green) are in close proximity to each other and sit between regularly organized eyelash cilia (red arrowhead). At the lid margin (Fig. 1J), CK1^+^ cells of the epidermis (orange) define the mucocutaneous junction as the transition to CK6^+^ conjunctiva (green) posterior to meibomian gland orifices (white arrow). In contrast, ICT of the 2-year old mouse eyelid reveals migration of the mucocutaneous junction to the level of meibomian gland orifices (Fig. 1K, white arrow). We also observed prominent truncation or atrophy of meibomian glands in the old lid (Fig. 1L). These glands have become largely obscured from view by eyelash cilia and are positioned much further apart from each other compared to the younger lid.

**Figure 2:**
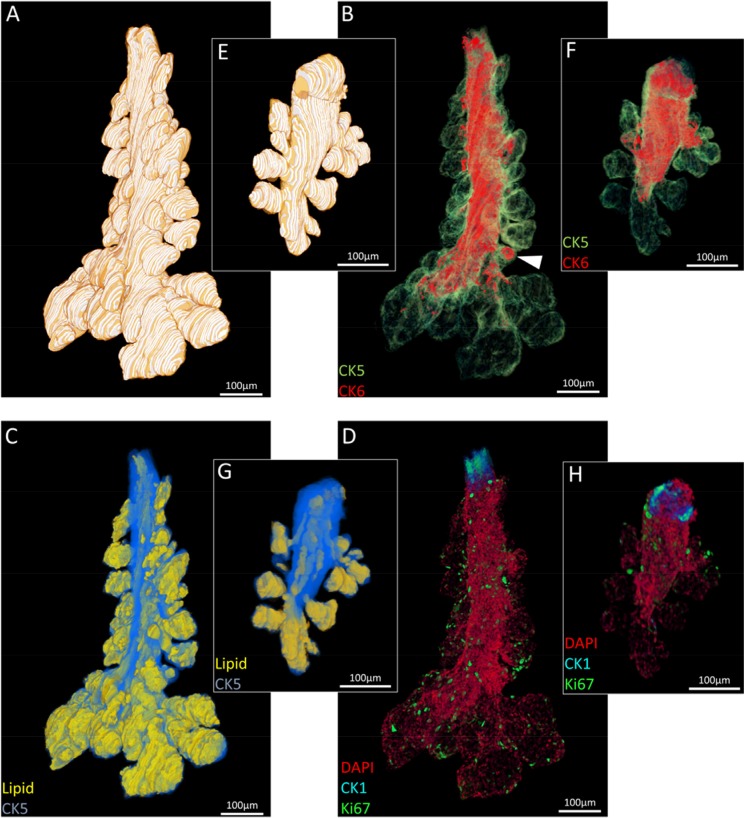
Segmented ICT reconstructions of (**A-D**) a 5month and (**E-H**) 2 year old mouse meibomian gland. In older meibomian glands, there is a notable loss of acini (**A:E**), ductal epithelium (**B:F**), lipid volume (**C:G**) and total cell number (**D:H**).

**Figure 3:**
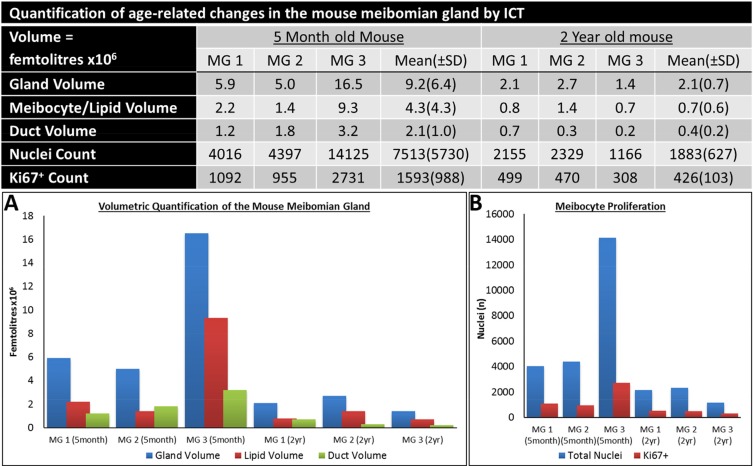
Quantification of the age-related changes in the Mouse meibomian gland by ICT.

Three meibomian glands were segmented from each eyelid reconstruction for visualization (Fig. 2) and quantification (Fig. 3) of total gland, ductal and lipid volumes as well as quiescent and proliferative cells. The 5-month old mouse meibomian glands (Fig. 2A-D) are comprised of multiple, large acini branching from a long tubular central duct characterized by a CK6^+^ epithelium (Fig. 2B). There are instances where CK6^+^ cells extend into an acinus (Fig. 2B, white arrowhead), implying that, in some cases the ductule, or terminal ends of the ductal epithelium, can form a significant component of an acinus. Acini are composed of terminally differentiated meibocytes filled with lipid (Fig. 2C) and Ki67^+^ cycling cells in the peripheral basal layer (Fig. 2D).

In comparison, an aged meibomian gland (Fig. 2E-H) had distinct shortening from the distal end and a marked loss in glandular and ductal volume (Fig. 2F) which reveals both ductal and acinar atrophy. Moreover, there is a visible reduction in the CK6^+^ ductal component of the gland (Fig. 2F). There is a clear loss in acinar tissue and acini appear noticeably smaller when compared to the younger glands (Fig. 2G). Ki67^+^ nuclei count is also largely reduced in the older meibomian gland and correlates with the loss of tissue and total cell number (Fig. 2H). The reduction of total gland, lipid and ductal volume (Fig. 3A), as well as a loss of quiescent and cycling cells (Fig. 3B) in the meibomian gland with aging was confirmed by ICT quantification.

### Keratinization of the Mouse Meibomian Gland with Age

**Figure 4:**
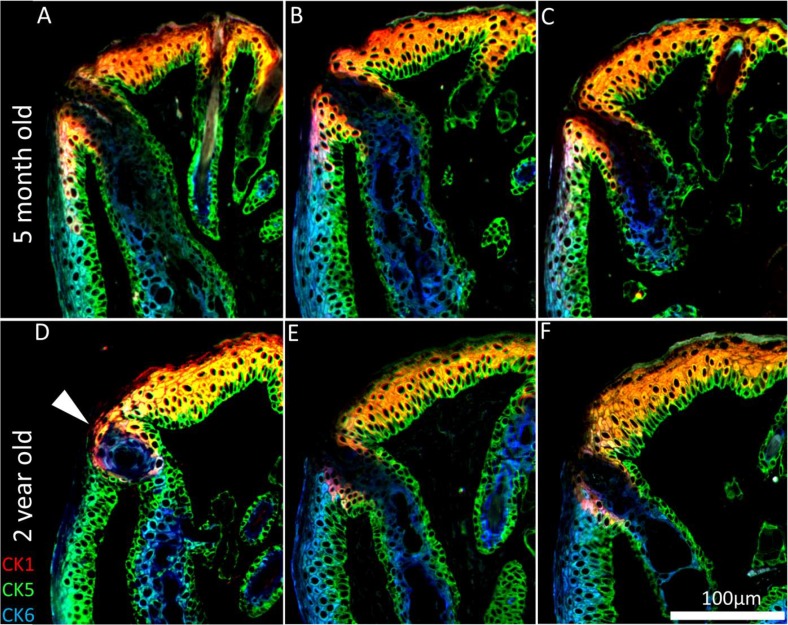
Keratinization of the meibomian gland orifice. (**A-C**) A 5month and (**D-F**) 2year old mouse lid margin cross-sections. (**D**) Gland plugging and (**D-F**) a loss of CK1^+^ cells posterior to the orifice causes the mucocutaneous junction to shift anteriorly in the aged mouse eyelid. CK1 immunostaining shows no hyper-keratinization or obstruction in the atrophic meibomian glands.

To assess whether hyper-keratinization of the meibomian gland duct and excretory orifice is associated with ARMGD in mice, ICT was specifically performed with CK 1, 5 and 6, markers for epidermis, all epithelium and meibomian gland duct/palpebral conjunctiva, respectively. In the young mouse eyelid, CK1 expression is limited to the suprabasal layer of the epidermis and extends posterior to the orifice of the meibomian gland (Fig. 4A-C). In older mice, hyper-keratinization of the duct and excretory orifice is not evident and does not correlate with glandular atrophy seen here. CK1 expression is less prominent at the lid margin resulting in an anterior shift of the mucocutaneous junction (Fig. 4D-F). In addition to lid margin changes, there are cases of meibomian gland orifice abnormalities in the form of CK5^+^/CK6^+^cell plugs (Fig 4D, white arrowhead).

**Figure 5:**
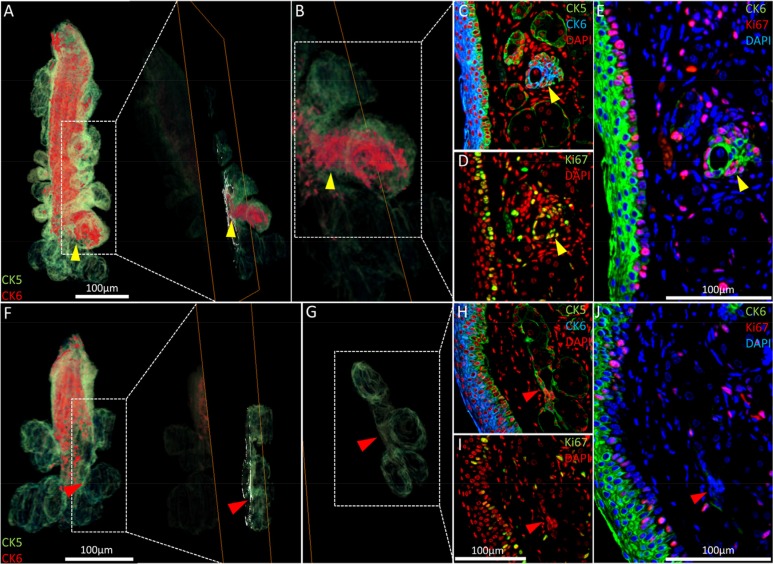
Nascent acini and their absence with aging. (**A**) A 3-D reconstruction of the 5 month old meibomian gland and an orthoslice taken at the region of interest: the transition between duct and acini (yellow arrowhead). (**B**) An acinus with a significant CK6^+^ component (**C**) also exhibits localized proliferation in the same region, according to Ki67 staining (**D**) and the resulting overlay (**E**). (**F**) The 3-D reconstruction of a 2 year meibomian gland with ductal truncation was evaluated at the same transitional region between duct and multiple acini (**G** - red arrowheads) and no prominent CK6 (**H**) or (**I**) Ki67 staining was evident when overlaying the sequential immunostains (**J**), suggesting a loss of proliferation in these zones.

As shown previously (Fig. 2B, white arrowhead), acini of the 5-month old meibomian glands may have an enlarged CK6^+^ epithelial component not readily apparent in meibomian glands reconstructed from the 2-year old mouse. Reverting to the same regions in the high-resolution 2-D images that constitute the reconstruction (Fig. 5A-E, yellow arrowheads), we found increased cell cycling when compared to the average acinus (Fig. 5D and E). These ‘nascent’ acini which contain a CK6^+^ ductal component contain fewer terminally differentiated meibocytes than acini with little or no CK6. Notably, no acini with a significant CK6^+^ ductal component were observed in the 2 year old meibomian gland (Fig. 5E-J). In the older glands, the ductal epithelium is truncated and the transitional regions between ductal and acinar epithelia show few cycling cells (Fig. 5H and J).

## DISCUSSION

Through ICT, we have visualized the aged related changes that occur in the mouse eyelid and meibomian glands three-dimensionally. At the lid margin, the mucocutaneous junction sets the boundary where the tear film meniscus meets dry skin and is maintained to facilitate normal outflow in the lacrimal functional unit. In the aged mouse eyelid, we observed an anterior migration of epidermis and the mucocutaneous junction to the level of meibomian gland orifices. This border is defined in humans as Marx's line by Lissamine green staining [33, 34]. The results presented here correlate with clinical studies which show increasing irregularities in the mucocutaneous junction and the position and width of Marx's line with aging [1, 33-36]. It is thought that perturbations of Marx's line may affect the volume or radius of curvature of tear menisci, which are symptomatic changes of aqueous-deficient dry eye [37, 38]. In the mouse model of ARMGD, we confirmed a loss of CK1^+^, epidermal cells posterior to meibomian gland orifices which results in forward migration of the mucocutaneous junction and presumably, conjunctivalization of the lid margin. As the anterior displacement of the mucocutaneous junction is proposed to affect Marx's line and lipid flow onto the ocular surface [34], the 2year old mouse may be a valuable model in studying the effects of an altered mucocutaneous junction on tear film mechanics and dry eye symptoms.

The conventional wisdom regarding the development of ARMGD is that obstruction and subsequent atrophy is caused by hyper-keratinization of the meibomian gland[11]. When quantifying young and old mouse meibomian glands through ICT (Fig. 3), we observed a marked decrease in total gland, duct and meibocyte/lipid volume in aged mice, confirming the atrophic effect of aging on the meibomian glands. From this data, we can expand on the acinar atrophy previously documented [26, 27, 30], to now associate both structural components of the gland, the acini and duct, with age-related atrophy. In mice, atrophy of both acinar and ductal tissue resulted in gland dropout comparable to human age-related gland dropout [4, 28, 30]. Our 3-D data suggests that age-related meibomian gland atrophy occurs in the absence of hyper-keratinization of the duct or orifice, as evidenced by the lack of CK1^+^ staining in these regions. Mice do show orifice plugging but with CK5^+^/CK6^+^ cells and not CK1^+^ cells. This is consistent with findings in humans that CK1/10 protein is not present in meibomian gland excreta [25] and suggests plugging occurs independent of hyper-keratinization. In older glands, orifice plugging could potentially result in obstructive MGD, however, gland atrophy exists with and without orifice plugging (Fig. 4), which suggests an unidentified trigger of glandular atrophy.

Based on observations of the ICT reconstructions of young mouse meibomian glands, a healthy gland contains ‘nascent’ acini that exhibit a significant CK6^+^ ductal epithelium component, or a thickened ductule, when compared to the average acinus. These potentially formative acini contain a high density of Ki67^+^ actively cycling cells and few terminally differentiated meibocytes. This suggests turnover of acinar tissue and may explain the varying acinar volumes observed within each meibomian gland (Fig. 2). The Ki67^+^/CK6^+^ region present in acini of young glands may potentially represent a transiently amplifying pool of progenitors that arise from asymmetric divisions of a nearby stem cell population, proposed to exist in the periphery of acini [39] or in the central duct [40]. These progenitors proliferate to form the acinar basal layer before becoming mature meibocytes, similar to the adult stem cell niche in hair follicles [41, 42] and the corneal limbus [43]. This is comparable to what has been proposed for the localization of progenitors of basal acinar epithelial tissue in skin sebaceous gland by retroviral transfer [44] and BLIMP1^+^ expression[45], which involves a slow-cycling cell population in the transition zone between acini and follicle that is responsible for renewing of the acinar basal cell layer. Like the meibomian gland acini, these basal cells migrate centripetally in a time-dependant manner and disintegrate in a holocrine fashion upon terminal differentiation.

The absence of proliferative CK6^+^ regions together with atrophy of ductal and acinar tissue in the older mouse meibomian glands, may point to an absence or senescence of progenitor cells and decreased renewal of both basal acinar and ductal epithelial cells. Senescence of progenitors has been postulated to be a key aging mechanism to decrease regenerative potential and prevent oncogenesis [46]. Furthermore, tissue-specific atrophy is known to occur as a consequence of cellular senescence and stem cell exhaustion [47-49]. However, further studies are required to better understand meibocyte progenitors as the localization and characteristics of the meibomian gland stem cell niche are currently unknown.

In conclusion, hyper-keratinization is not considered to be a factor in ARMGD from observations of aged mice lids. Alternatively, age-related meibomian gland atrophy may result due to a loss of progenitors or meibocyte proliferation, specifically at the transitional region between duct and acini which, in young glands, may be largely comprised of CK6^+^ epithelia. The atrophy of meibomian glands would be expected to cause hypo-secretion of meibum on the ocular surface whereas the relocation of the mucocutaneous transition would be expected to affect tear film dynamics to promote the evaporation of tears. As a result, future therapies to evaporative dry eye may have to focus on regenerative medicine or pharmaceutics to combat the decline of meibocyte progenitors, meibomian gland atrophy and the loss of CK1^+^ epidermal cells at the mucocutaneous junction with age.

## METHODS

### Sample Processing

Animals were treated according to the ARVO statement on the use of animals in vision research and experiments were approved by the IACUC of the University of California, Irvine (P.I. Jester, protocol# 2011-3002, approved September 8, 2011). A 5month and 2 year old mouse were selected as appropriate subjects based on previous research which identified when age-related meibomian gland changes occur [27]. For the young mice, the glands were collected from healthy mice with no gross eyelid or ocular defects. Mice were sacrificed through carbon dioxide asphyxiation and immediate cervical dislocation and eyelids were excised and treated overnight with 2% paraformaldehyde in phosphate buffered saline (PBS). After fixation, the eyelids were embedded in low melting point 3% agarose to orientate the tissue for sectioning and then dehydrated in an ascending ethanol series (50-75-100%) over 3hrs. The embedding media used, Butyl-Methyl Methacrylate (BMMA), was formulated to enable sectioning of relatively soft tissues and all embedding was carried out at 4°C. BMMA infiltration was carried out with decreasing concentrations of ethanol:BMMA (2:1,1:1,1:2) before overnight rotation in 100% BMMA and polymerization under UV light at 4°C for 10hrs in gelatin BEEM capsules.

### Serial Sectioning and Sequential Immunostaining

For 3-D reconstructions of mouse eyelids, sections were cut serially with a diamond knife on a Reichart Ultramicro-tome at 2μm to achieve a high axial resolution while enabling the physical cutting of 1.3mm (5-month) and 1.1mm (2-year) of tissue. Sections lost in the sectioning or immunostaining process can effectively be replaced by interpolation of an adjacent image with the defective image to average and fill in the missing data computationally. In this experiment, 3% (17 sections) of the young and 2% (11 sections) of the old mouse eyelid sections were replaced with averaged data as they were damaged in the staining process. After sectioning and adherence to gelatin-coated microscope slides, sections were sequentially immunostained in a TedPella Biowave microwave and antibody dilutions used in this experiment are detailed in Supplemental Table 1. Immunostaining procedures for ICT have been previously described [31]. Elution of antibodies from the section for sequential immunostaining was achieved with glycine hydrochloride pH 2.0 heated to 50°C for 1hr.

After immunostaining, sections were mounted in 50:50 Glycerol:PBS with 1:10,000 4'-6-Diamidino-2-phenylindole (DAPI) to identify cell nuclei. The only secondary antibody used in this experiment was goat anti-rabbit AlexaFluor 546 to demonstrate removal of sequential antibodies.

### Fluorescent Imaging and 3-D Reconstruction

For fluorescent imaging of serial sections, a Leica DMI6000B equipped with an ASI automated stage and programmed with metamorph software was used. In our case, the slide holder accommodates 4 microscope slides which hold approximately 50 sections per slide at a thickness of 2μm, allowing approximately 400μm of tissue depth to be imaged per scan. Eyelid sections were imaged with a Leica 20× 0.7NA objective in 3×3 mosaics with a pixel size of 0.46μm × 0.46μm, stitched together using metamorph software (Molecular Devices, Sunnyvale CA). The final montage image becomes a 2-D plane that is aligned with montages of adjacent sections as part of a series of images in the final 3-D reconstruction. The montages were made into a stack in ImageJ [50] and semi-automatically aligned in the Amira 5.4 software package (Visage Imaging, San Diego CA). Volume rendering and segmentation was also carried out in Amira. The CK5 and 6 volume reconstructions were used to quantify the gland and ductal volume while the meibocyte/lipid component was measured by rendering the low intensity pixels (<5) that are a result of a loss of keratin in the cytoplasm of differentiated meibocytes or lipid extraction in the dehydration stage of the embedding process. Segmentation of the entire gland involves tracing the contours of the basal layer visualized by CK5 in each image that makes up the 3-D reconstruction. Total gland and lipid volumes were then measured though ImageJ by converting total voxel number into a volume by multiplying the known volume of a single voxel (0.4232μm^3^) with total voxel count. To quantify cell nuclei and Ki67^+^ nuclei, the 3-D objects counter plugin was used in ImageJ. These cell counts were validated with physical counting for accuracy.

## SUPPLEMENTARY DATA


