# Supplementary material for: Absence of ductal hyper-keratinization in Mouse age-related meibomian gland dysfunction (ARMGD)
Source: Aging (Albany NY). 2013 Nov 18;5(11):825–34. doi: 10.18632/aging.100615 (PMC3868725; doi:10.18632/aging.100615)
Supplement: Supplementary file 1 [file aging-05-825-s001.pdf]

Antibody	Vendor	Concentration	Dilution
**Rb Anti-Ki67**	Abcam	0.2 mg/ml	1/250
**Rb Anti-Cytokeratin 1**	Abcam	1 mg/ml	1/2500
**Rb Anti-Cytokeratin 5**	Abcam	1 mg/ml	1/2500
**Rb Anti-Cytokeratin 6**	Abcam	1 mg/ml	1/2500
**AlexaFluor 546 Secondary**	Invitrogen	2 mg/ml	1/1000 (ki67) 1/4000 (CKs)
